# Timing and factors associated with first antenatal care booking among pregnant mothers in Gondar Town; North West Ethiopia

**DOI:** 10.1186/1471-2393-14-287

**Published:** 2014-08-25

**Authors:** Temesgen Worku Gudayu, Solomon Meseret Woldeyohannes, Abdella Amano Abdo

**Affiliations:** Department of Midwifery, College of Medicine and Health Sciences, University of Gondar, Gondar, Ethiopia; Department of Epidemiology and Biostatistics, College of Medicine and Health Sciences, University of Gondar, Gondar, Ethiopia

**Keywords:** Timing at first antenatal care booking, Gondar Town

## Abstract

**Background:**

Antenatal care service which is among strategies to maintain maternal and fetal wellbeing is strongly recommended to be initiated early during pregnancy. To developing world where there is uncommon practice of pre-pregnancy care and support, timely commencement is crucial in getting potential benefits from some of the elements of the care. Therefore, we sought to assess timing and factors associated with the first antenatal care booking among pregnant mothers attending antenatal care clinics in Gondar town health facilities; North West Ethiopia.

**Methods:**

Health institution based cross-sectional study was conducted among pregnant mothers from April to June 2012 in Gondar town. A total of 407 pregnant mothers were interviewed at exit from antenatal clinic by using structured and pre-tested questionnaire. Bivariate and multivariate data analysis was performed using SPSS for Windows version 16.0.

**Result:**

The study indicated that 35.4% of mothers started antenatal care timely (in the first trimester of pregnancy). The mean time was 4.5 months (17.7 weeks) of pregnancy. Multivariate logistic regression analysis showed that: [(AOR (95% CI)) maternal age ≤ 25 (1.85 (1.10, 3.09)), age at marriage ≥20 years (2.21 (1.33, 3.68)), pregnancy recognition by urine test (2.29 (1.42, 3.71)), mothers who perceived the right time to start antenatal care within first trimester (3.93 (2.29, 6.75)) and having decision power to use antenatal care (2.43 (1.18, 4.99))] were significantly associated with timely commencement to antenatal care.

**Conclusion:**

Timely entry to antenatal care was low in the study area. In order to improve the situation, it is important to provide community based information, education and communication on antenatal care and its right time of commencement. In addition, empowering women and implementing the proclamation designed for the age at marriage is mandatory up to the local level.

**Electronic supplementary material:**

The online version of this article (doi:10.1186/1471-2393-14-287) contains supplementary material, which is available to authorized users.

## Background

While motherhood is often a positive and fulfilling experience; for many women it is associated with suffering, ill-health and even death. Worldwide up to 358,000 women die each year in pregnancy and childbirth. Of this, 99% of maternal deaths occur in developing countries where 85% of the populations live. More than half of these deaths occurred in sub-Saharan Africa. In Ethiopia, maternal mortality and morbidity levels are among the highest in the world. The Maternal Mortality Ratio (MMR) in the year 2011 was 676 per 100,000 live births [1–3].

The antenatal period provides excellent opportunities to reach pregnant women with preventive and curative care. It is revealed that, the higher the level of care obtained during pregnancy, the higher the use of safe delivery service will be. This strong positive association between level of care obtained during pregnancy and the use of safe delivery care might help explain why antenatal care could also be associated with reduced maternal mortality [4, 5].

The new WHO antenatal care model and the national institute for health and clinical excellence guidelines recommend that first antenatal care (ANC) booking should occur within the first trimester of pregnancy. Among several examinations and tests recommended by WHO; timely booked mothers will be offered screening for HIV infection which helps early detection and prevention of transmission [6–8]. Moreover, women in their ANC visit will get a screening for syphilis at an early stage of pregnancy and treated as early as possible. In untreated maternal syphilis 70%-100% of infants will acquire the infection and one third will be stillborn [9].

More than half of all pregnant mothers experience nausea and vomiting that might occur at any time of day or night usually from 4–7 weeks of pregnancy. It might alter maternal nutritional needs and results in craving of non-nourish substances and un-prescribed medications which potentially have teratogenic effects unless otherwise they receive counseling during their early pregnancy [7, 10]. On the other hand provision of micronutrient supplements especially iron and folic acid during early pregnancy and if possible before conception is among strongly recommended interventions. It is well known that folate deficiency in early pregnancy is associated with congenital malformations such as neural tube defects and increased DNA damage. Besides such birth defects, babies of mothers with folate deficiency are more likely to be small for gestational age, delivered pre-term, develop sever language delay and even are at high risk for mortality [11–14].

While there are potential benefits to be gained from some of the elements of ANC and these benefits are most crucial for developing countries where maternal morbidity and mortality levels are high; the recommended first visits by skilled providers is received by few pregnant women in those countries [14]. Though basic ANC is provided for free in Ethiopia, according to Demographic and Health Survey 2011, only 34% and 11.2% of women made at least one visit and their first ANC visit before the fourth month of pregnancy respectively [3]. The aim of this study therefore was to find out the proportion of women who were booked at the recommended time and identify factors contributing for timely entry to ANC.

## Methods

Health institution based cross-sectional study was carried out in Gondar town; the capital of North Gondar zone, from April to June 2012. The town is 741 Km North West to Addis Ababa, the capital of Ethiopia and 180 Km North to Bahir Dar, the capital of Amhara regional state. In the town there are five health centers and one referral teaching hospital owned by the government. The town has 13 urban and 11 rural administrative kebeles^a^ with a projected total population of 248,784 in the year 2010/2011 [15]; of which 131,111 were females.

The included participants in the study were all pregnant mothers who came for ANC visit. Mothers who were sick and who didn’t remember their last normal menstrual period were excluded.

The sample size was determined by using a single population proportion formula which took the following assumptions in to consideration: the proportion of mothers who entered in to ANC timely 40.2% [16], 5% level of significance (α = 0.05) and 5% margin of error (ω = 0.05). The final sample size was adjusted by adding 10% non response rate and thus turned out to be 407. Information about the client flow to each health institution was obtained from Gondar town health department [17] and based on it; skip interval was calculated. Since all mothers were pregnant and received the same focused antenatal care, systematic random sampling technique was used to select the study subjects and every third client was selected and included in the study.

Data were collected through face to face interviews using a structured and pre-tested questionnaire. The questionnaire was first prepared in English then translated to Amharic and back to English again by language expert in order to maintain the consistency of the instrument. A pre-test was conducted on 20 pregnant mothers in one of the health centers out of the study area called Makisegnit Health Center before the main and the instrument was amended accordingly. Seven diploma nurses had conducted the face to face interviews and two BSc. degree Midwives had supervised the data collection process. Training was given to the data collectors and supervisors before the actual data collection regarding the aim of study, data collection tool and procedures.

The information was collected on mothers’ age, mother’s age at marriage, marital status, place of residence, family income, educational status, occupation, educational status of the husband, occupation of the husband, age difference between the mother and the husband, distance from health facility, family size; obstetrics variables such as: previous ANC visit, parity, gravidity, history of abortion and still birth, means of pregnancy recognition, awareness of timing at first ANC booking. In addition data on mother’s ability of having decision power to use ANC service was included (Additional file 1).

In this study gestational age means the age of the fetus in weeks from the last normal menstrual period of the mother. Timely antenatal care booking is booking to ANC service before 12 complete gestational weeks. Decision to use ANC in this study is also defined as Mothers ability of deciding by themselves or jointly with their partner to utilize current ANC.

Data entry was done by using EPI Info version 3.4.3 and exported to SPSS version 16.0 software package for analysis. The data first was analyzed using bivariate logistic regression and then all explanatory variables which had p-value ≤ 0.2 were entered into multivariate logistic regression model to determine the effect of various factors on the outcome variable and to control confounding effects. The results were presented in the form of tables, figures and texts using frequencies and summary statistics such as median, mean, standard deviation and percentage to describe the study population in relation to relevant variables. The strength of association between independent and dependent variables was assessed using the odds ratio with 95% confidence interval.

Ethical clearance was obtained from the institutional review board of University of Gondar. A formal letter request of cooperation was written to Gondar town health office. Verbal consent was obtained from each study participants. Confidentiality of information and privacy was maintained. Finally, the research was checked for the adherence to STROBE guidelines (Additional file 2).

## Results

### Socio demographic characteristics of the study population

The study included a total 407 pregnant mothers out of which 85% were aged 20–35 years with a median age of 25 years. Orthodox Christianity was the dominant religion among respondents and 97.5% were Amhara by ethnicity. Married mothers were nearly 94% and about one third had attended secondary school and above (Table 1).

**Table 1:** **Percentage distribution of the study population by selected socio demographic characteristics; Gondar Town, Ethiopia, April-June 2012**

Variables	Number (n = 407)	Percentage
**Maternal age**		
< 20	40	9.8
20–35	346	85.0
> 35	21	5.2
**Marital Status**		
Single	15	3.7
Married	381	93.6
Other^***†***^	11	2.7
**Religion**		
Orthodox	358	88.0
Muslim	44	10.8
Other^***††***^	5	1.2
**Ethnicity**		
Amhara	397	97.5
Other^***†††***^	10	2.5
**Educational status**		
Unable to read and Write	147	36.1
Read and Write	45	11.1
Primary	84	20.6
Secondary and above	131	32.2

^***†***^ *Divorced, Cohabit* ^***††***^Catholic, protestant, Judaism ^***†††***^Tigrie, Oromo, “Bete-Israel”.

### Obstetric characteristics of the study population

Fifty six percent of mothers have been pregnant more than once and about 66% of them had used ANC previously. Almost half of the participants were recognized their pregnancy via urine test and the rest by missing menses. About 86% of mothers had decision power to use current ANC (Table 2).

**Table 2:** **Percentage distribution of the study population by selected Obstetric characteristics; Gondar Town, Ethiopia, April-June 2012**

Variables	Number (n = 407)	Percentage
**Parity**		
Nullipara	178	43.7
Para one and above	229	56.3
**Previous ANC use (n = 229)**		
Yes	150	65.5
No	79	34.5
**Means of pregnancy recognition**		
Missing period	203	49.9
Urine test	204	50.1
**Gestational Age at 1** ^**st**^ **ANC Booking**		
≤ 12 weeks	144	35.4
> 12 weeks	263	64.6
**Decision on current ANC use**		
Yes	348	85.5
No	59	14.5

### Pregnant mother’s commencement to ANC

**Figure 1:**
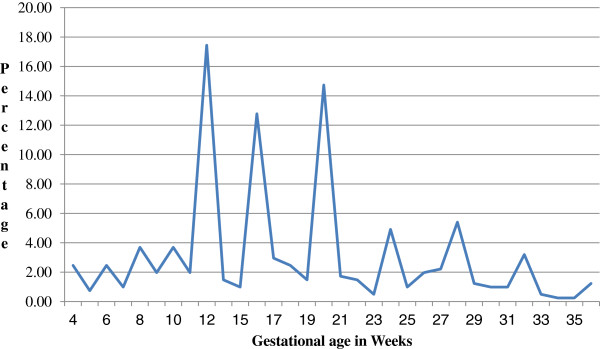
**Percentage of pregnant women by gestational age in weeks during ANC booking, Gondar town, Ethiopia, April-June 2012.**

This study identified that 35.4% of participants started their ANC timely (Table 2). The timing of first ANC booking ranged from 4 weeks to 36 weeks of pregnancy, the peak being at 12^th^ week of pregnancy. The mean gestational age during first antenatal care booking was 17.7 weeks with standard deviation 7.5 weeks. Majority of participants (64.6%) booked late and six of the mothers (1.5%) booked near to their time of delivery (Figure 1).

**Figure 2:**
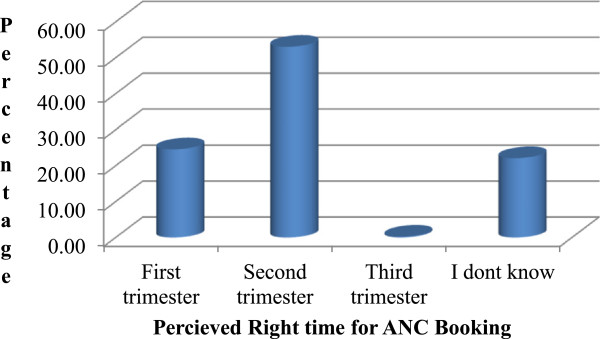
**Percentage of pregnant women who perceived the right time for ANC booking, Gondar town, Ethiopia, April-June 2012.**

Concerning pregnant mother’s perception of right time for ANC booking; about quarter of respondents answered the right time to be within the first three months of pregnancy while 53.3% still perceiving the right time to be beyond three months. There were also 22.1% of mothers they didn’t know when to commence ANC (Figure 2).

### Determinants of timely booking for ANC

The result of Logistic regression analysis showed that pregnant mothers aged 25 and below were nearly two times more likely to commence ANC within the recommended time compared to their counter parts (adjusted OR [95% CI] = 1.85 (1.10, 3.09)). Likewise mothers whose age at marriage above twenty years were two times more likely to start their ANC within the first three months of pregnancy than those who married during their teens (adjusted OR [95% CI] = 2.21 (1.33, 3.68)). Urine test as a means of pregnancy recognition and having decision power to use ANC service among mothers were significantly associated with timely booking for ANC (adjusted OR [95% CI] = 2.29 (1.42, 3.71) and 2.43 (1.18, 4.99)) respectively. Similarly perceived right time within three months of pregnancy was significantly associated (adjusted OR [95% CI] = 3.93 (2.29, 6.75)) (Table 3). Whereas variables like: maternal level of education, wealth index, parity, residence and maternal health status showed no significant association.

**Table 3:** **Logistic Regression analysis of socio-demographic and Obstetric factors for timing at first ANC booking; Gondar town, Ethiopia, April-June 2012**

Variables	Timing at first ANC booking in weeks		COR (95% CI)	AOR (95% CI)
	Timely (≤12 Wks)	Late (>12Wks)		
	No. (%)	No. (%)		
**Maternal age/years**				
◦ ≤ 25	66 (41.8)	92 (58.2)	1.57 (1.04,2.38)*	1.85 (1.10, 3.09)*
◦ > 25	78 (31.3)	171 (68.7)	1	1
**Marital status**				
◦ In Marriage	130 (34.1)	251 (65.9)	1	
◦ Out Marriage	14 (53.8)	12 (46.2)	2.25 (1.01, 5.01)*	NS
**Age at marriage**				
◦ <20	63 (29.4)	151 (70.6)	1	1
◦ ≥20	81 (42.0)	112 (58.0)	1.73 (1.15, 2.61)*	2.21 (1.33, 3.68)*
**Mothers Occupation**				
◦ House wife	86 (31.7)	185 (68.3)	1	
◦ Employed	49 (42.2)	67 (57.8)	1.57 (1.01, 2.46)*	NS
◦ Daily laborer	9 (45.0)	11 (55.0)	1.76 (0.70, 4.41)	
**Maternal Educational Status**				
◦ Unable to read and write	41 (27.9)	106 (72.1)	1	
◦ Read and Write	12 (26.7)	33 (73.3)	0.94 (0.44, 1.99)	NS
◦ Primary education	39 (46.4)	45 (53.6)	2.24 (1.28, 3.92)*	
◦ Secondary and above	52 (39.7)	79 (60.3)	1.70 (1.03, 2.81)*	
**Family size**				
◦ ≤ 3	108 (40)	162 (60)	1.87 (1.19, 2.94)*	NS
◦ > 3	36 (26.3)	101 (73.7)	1	
**Parity**				
◦ Primipara	75 (42.1)	103 (57.9)	1.69 (1.12, 2.55)*	NS
◦ Multipara	69 (30.1)	160 (69.9)	1	
**Pregnancy recognition**				
◦ Missing period	49 (24.1)	154 (75.9)	1	1
◦ Urine test	95 (46.6)	109 (53.4)	2.74 (1.79, 4.18)**	2.29 (1.42, 3.71)*
**Get information when to book**				
◦ Yes	96 (40.3)	142 (59.7)	1.70 (1.12, 2.60)*	NS
◦ No	48 (28.4)	121 (71.6)	1	
**Perceived right time to book**				
◦ I don’t know	33 (36.7)	57 (63.3)	1.63 (0.96, 2.75)	3.55 (1.79, 7.05)**
◦ ≤ 12 weeks	54 (54.0)	46 (46.0)	3.29 (2.01, 5.41)**	3.93 (2.29, 6.75)**
◦ >12 weeks	57 (26.3)	160 (73.7)	1	1
**Decision on current ANC use**				
◦ Yes	132 (37.9)	216 (62.1)	2.39 (1.23, 4.68)*	2.43 (1.18, 4.99)*
◦ No	12 (20.3)	47 (79.7)	1	1

*p-value <0.05 **p-value <0.001.

NS = Variables not associated by back ward step wise logistic regression analysis.

n = 407.

## Discussion

According to WHO and the National Institute for Health and Clinical Excellence recommendations; every pregnant mother should start ANC booking during the first trimester of pregnancy [9, 10]. However in this study only 35% of mothers registered for ANC at the right time and the rest 65% booked late. The proportion of pregnant mothers who booked within the recommended time was low in this study compared with the finding from Addis Ababa [16]; this difference could be due to the fact that, Addis Ababa is the capital of the country and the community there might have better health awareness than other parts of the country. On the other hand the finding in this study was higher than compared with study from national finding [3], since majority of participants in national study were rural residents with late or no entry to ANC. Also, in other Africa countries [18–23] timely entry to ANC is lower than current study. The time gap between studies and socio-cultural differences among the study population could explain it.

The mean gestational age at which pregnant mothers booked the first ANC in this study was 17.7 weeks (4.5 months). This is comparable with previous study done in Addis Ababa in which the mean gestational age at booking was 4 months [16]. This might be explained by the current practice of starting ANC at fourth months of pregnancy that is being practiced widely since implementation of BEmONC manual and HMIS guide line [24], as it is stated in these documents that first ANC visit is better be started before 16 weeks of gestation.

In this study, mothers who are aged 25 years and below were about 2 times more likely to start ANC within the recommended time than those whose age was above 25 years. This finding is supported by studies done in Addis Ababa and Nigeria [16, 21]. The possible explanation could be younger pregnant mothers might more likely be literate than elder mothers. Alternatively, elder mothers might consider starting ANC at delayed time not as a problem from their previous experiences. On the other hand, pregnant mothers who were above twenty years at the time of marriage were near to 2 times more likely to be booked to ANC within first trimester of pregnancy than their counter parts. The explanation could be, the fact that awareness and level of decision making increases with age.

The current study also revealed that pregnant mothers who confirmed their pregnancy by urine test were about 2 times more likely to commence ANC within the first trimester of pregnancy. Similarly, Bivariate analysis showed that same association was found in the study from Addis Ababa [16], this indicates that urine tests in our situation is done in the institutions so that helping mothers to commence ANC at the time they came to confirm pregnancy.

Perception of the mothers regarding timing had as well statistically significant association with timely entry to ANC. Mothers who perceived the right time to be in the first trimester were nearly 4 times more likely to commence ANC timely than those who perceived the right time beyond 12 weeks of pregnancy. This finding is agreed with the study from Addis Ababa. This entails that respondents who were informed to book within 12 weeks of gestation were more likely to book within the recommended time [16]. Moreover, it is in line with the study conducted in Nigeria which suggested that pregnant mothers who have knowledge were booked timely [22]. This put forward that provision of proper information and advice on the patterns of ANC utilization from service providers improves timely registration to ANC.

Women involvement in the decision making process is crucial and help mothers to utilize maternal health service which directly and indirectly reduces maternal morbidity and mortality. As observed in this study, mothers who decided to use ANC by themselves and jointly with their husband were more than two fold to be booked in the right time than un-deciding mothers. Taking part in the decision making process helps the mother to get support from husband that in turn increases utilization of maternal health service. This is also supported by study done in Tanzania [23], in which not being supported by the husband or partner were identified as factors associated with a later antenatal care enrolment. This in other ways indicates the more support from the husband, the timely commencement to ANC would be.

The variables used in the study might not be exhaustive and some other variables might be missed such as: timing of all previous ANC utilization, birth to pregnancy interval, timing for different age groups especially adolescents and reasons for timely and late entry to ANC which might affect the current timing at first ANC booking. In addition, qualitative and large scale community based studies that can address none ANC users should be conducted.

## Conclusion

Only one third of pregnant mothers in this study practiced timely booking of first ANC. Current age of the mother 25 years and below, age at marriage above 20 years, means of pregnancy recognition via urine test, perceived right time as first trimester and having decision power to in using current ANC were statistically significant factors for timely booking. It is important to provide community based information, education and communication on antenatal care and its right time of commencement. In addition, empowering women and strengthening and implementing the proclamation designed for the age at marriage is mandatory up to the local level.

## Endnote

^a^Kebele is the smallest administrative unit of the Federal Democratic Republic of Ethiopia.

## Electronic supplementary material

Additional file 1: **English questionnaire.** (PDF 186 KB)

Additional file 2:: **STROBE Statement check list.** (PDF 68 KB)

## Abbreviations

ANC: Antenatal Care
BEmONC: Basic, Emergency Obstetric & Neonatal Care
HMIS: Health Management Information System.
