# Supplementary material for: Timing and factors associated with first antenatal care booking among pregnant mothers in Gondar Town; North West Ethiopia
Source: BMC Pregnancy Childbirth. 2014 Aug 25;14:287. doi: 10.1186/1471-2393-14-287 (PMC4152591; doi:10.1186/1471-2393-14-287)
Supplement: Supplementary file 1 — Additional file 1: English questionnaire. (PDF 186 KB) [file 12884_2013_1160_MOESM1_ESM.pdf]

10.1 Annex -I- English questionnaire

University of Gondar College of Medicine and Health Sciences

Department of Midwifery

Questionnaires to assess the timing at first antenatal care booking and associated factors among pregnant mothers attending ANC clinics at health institutions in Gondar town, North West Ethiopia.

Hello, my name is _______________ and I am staff in this health facility and working with Temesgen Worku, MSc. Midwifery student at University of Gondar. As part of this survey, we are collecting information on timing at first antenatal care booking and associated factors among pregnant mothers attending ANC clinics at health institutions in Gondar town. We will include all mothers who came for usual ANC visit. The survey will take about 25 minutes.

The questionnaire includes socio-demographic and obstetric factors. All information you provide will be kept confidential. I will not include any identifiers, such as you name or exact address. Only honest answers would contribute for improvement of health planning. Your role in the success of the research is important and I appreciate your contribution to the research. Would this be okay for you?

I understood about the advantage of the research and the roles I will have in the research. I have agreed to participate in the research. **A. Yes** **B. No**

If respondent agrees to be interviewed,

Starting time ___________________: End time: _________________

Thank you for your participation!

Date of data collection ____

Name of data collector ________________________ Signature ________________

Name of supervisor __________________________ Signature ________________

Section 1: Socio-demographic information

Q.No	Question	Code	Go To Q
101	Age	-------------- Years	
102	Marital status	01. Single<br>02. Cohabit<br>03. Married<br>04. Widowed<br>05. Divorced	**If not married Go to 106**
103	If married; type of marriage	01. Arranged<br>02. Love<br>97. Others(specify) -------------	
104	If married; Age at marriage	------------ Years	
105	Age difference between you and your husband	----------------- Years	
106	Residence	01. Urban<br>02.Rural	
108	Religion	01. Orthodox<br>02. Catholic<br>03. Protestant<br>04. Muslim<br>97. Others(specify) -------------	
109	Ethnicity	01. Amhara<br>02. Tigrie<br>03. Oromo<br>97. Others(specify) -------------	
110	Occupation	01. House wife<br>02. Government employee<br>03. Privet employee<br>04. Privet business<br>97. Others(specify) -------------	
111	Educational status	01.---------------- class completed<br>02.College/University completed	
112	What is your profession?	01. Health science<br>02. Education<br>03. Agriculture<br>04. Business & Social sciences<br>05. Non health natural sciences<br>97. Others (Specify) __________	
113	Monthly house hold income in Ethiopian Birr	_____________________Birr	
114	How far your home from this institution?	_____________________Km	
115	Family Size	____________________________	

Questions Number 116-120 will be asked if the answer for question number 102 is married/cohabit.			
116	Husband's age	--------------	
117	Husband's occupation	01. Farmer<br>02. Government employee<br>03. Private employee<br>04. Private business<br>97. Other; specify _________	
118	Husband's Religion	01. Orthodox<br>02. Catholic<br>03. Protestant<br>04. Muslim<br>97. Others(specify)	
119	Husband's educational status	01. ---------------- class completed<br>02. College/University completed	
120	What is your husband's profession?	01. Health science<br>02. Education<br>03. Agriculture<br>04. Business & Social sciences<br>05. Non health natural sciences<br>97. Others (Specify) _________	

Section 2: Obstetrics Related Information

Q.No	Question	Code	Go To Q
201	Is it your first time pregnancy?	01. Yes<br>02. No	**If yes Go to 212**
202	How many times in total you became pregnant?	____________	
203	How many times in total you gave birth?	____________	
204	How many of your pregnancies resulted in a baby that was born alive?	____________	
205	How many of your pregnancies resulted in a baby that was born dead?	____________	
206	Is any of your previous pregnancy ended with abortion?	01. Yes<br>02. No	
207	Have you had antenatal care for the past pregnancies?	01. Yes<br>02. No	
208	Was there any complication in previous pregnancy/ies?	01. Yes<br>02. No	
209	If Yes for Q-208, what was it?	01. Vomiting<br>02. Sever head ache<br>03. Decrease fetal movement<br>04. Amniotic fluid leakage<br>05. Vaginal bleeding<br>06. Abortion<br>07. Hypertension<br>97. Others, specify --------	

Section 2: Obstetrics Related Information

Q.No	Question	Code	Go To Q
210	Have you had any complication during previous labor and delivery/ies?	01. Yes<br>02. No	
211	If yes, what was it?	01. Pre-term labor<br>02. Prolonged labor<br>03. Fetal death/still birth<br>04. Amniotic fluid leakage<br>05. PPH<br>06. Retained pregnancy<br>07. Puerperal sepsis<br>97. Others, specify --------	
212	Have you had any previous caesarean birth?	01. Yes<br>02. No	
**Current Pregnancy Related Questions**			
213	By what means you recognized that you are pregnant?	01. Missing period<br>02. Urine test<br>97. Other; specify______	
214	Is this pregnancy approved by your husband and family?	01. Yes<br>02. No	
215	Is this pregnancy planned?	01. Yes<br>02. No	
216	What is your financial source for this ANC service expenses?	01. My self<br>02. My husband<br>03. Me and my husband<br>97. Other; specify______	

Section 2: Obstetrics Related Information

Q.No	Question	Code	Go To Q
217	Have you had any one or more of the following complications during this pregnancy?	01. Vomiting<br>02. Sever head ache<br>03. Decrease fetal movement<br>04. Amniotic fluid leakage<br>05. Vaginal bleeding<br>06. Abortion<br>07. Hypertension<br>97. Others, specify --------	
218	For this pregnancy, at what gestational age you first booked to antenatal care.	___________ Months	
219	Why you booked during this/that period?	01. I thought it is the right time<br>02. Misconception of the right time and its purpose<br>03. I didn't know that I'm pregnant<br>04. Ignorance<br>05. Other, specify __________	
220	When do you think the appropriate time for first antenatal care booking?	At ___________ Months	
221	Did you get any information when to book to ANC?	01. Yes<br>02. No	
222	If yes for Q-221, what is/was the source of information?	01. Health workers<br>02. Friends<br>03. Media<br>97. Other, specify______	

SECTION 3: Questions used to asses Decision making status of women

S. No	Questions	Response	Code
301	Who is in your family usually has final say in the following decisions<br>1. Your own health care?<br>2. Using Current ANC<br>3. Making large household purchase eg<br>3.1. land,<br>3.2. constructing house,<br>3.3. cow,<br>3.4. changing residence<br>3.5. TV,<br>4. Making household purchase for daily need(salt, onione,….)<br>5. Visits to family, friends or relatives<br>6. What food should be cooked everyday<br>7. What to do if a family member become sick	01 0 2 03 97<br>Women alone Husband Jointly Others<br>______ ______ ______ ______<br>______ ______ ______ ______<br>______ ______ ______ ______<br>______ ______ ______ ______<br>______ ______ ______ ______<br>______ ______ ______ ______<br>______ ______ ______ ______<br>______ ______ ______ ______<br>______ ______ ______ ______<br>______ ______ ______ ______<br>______ ______ ______ ______	

Thank you very much!
