# Supplementary material for: Timing and factors associated with first antenatal care booking among pregnant mothers in Gondar Town; North West Ethiopia
Source: BMC Pregnancy Childbirth. 2014 Aug 25;14:287. doi: 10.1186/1471-2393-14-287 (PMC4152591; doi:10.1186/1471-2393-14-287)
Supplement: Supplementary file 2 — Additional file 2:: STROBE Statement check list. (PDF 68 KB) [file 12884_2013_1160_MOESM2_ESM.pdf]

Topic of study: Timing and factors associated with first antenatal care booking among pregnant mothers in Gondar Town; North West Ethiopia

ID. **1440044905913809**

STROBE Statement—Checklist of items that should be included in reports of ***cross-sectional studies***

	Item No	Recommendation
**Title and abstract**	1	(*a*) Study design is indicated in the abstract, methods section as a health institution based cross-sectional study design.
		(*b*) A balanced summary of what was done and what was found is provided in the methods and result section of the abstract
**Introduction**		
Background/rationale	2	Scientific background and rationale for the investigation is precisely reported here.
Objectives	3	Specific objectives are stated as the final sentence of last paragraph under background session
**Methods**		
Study design	4	It is presented in the first sentence of first paragraph of methods session.
Setting	5	A brief detail of the setting, locations and study period is described in the first paragraph as well.
Participants	6	(*a*) Eligibility criteria, the sources and methods of selection of participants are clearly stated in 2nd and 3rd paragraphs of methods session.
Variables	7	Both outcome and explanatory variables are defined in the 5th and 6th paragraphs of methods session.
Data sources/ measurement	8*	Source of data and data analysis methods are discussed in the 4th and 7th paragraphs of methods session.
Bias	9	Efforts to address potential sources of bias were described under 4th & 7th paragraphs of methods session.
Study size	10	How the study size was arrived at is explain in the 3rd paragraphs of methods session.
Quantitative variables	11	All quantitative variables treated as qualitative after categorizing them in one of most commonly used categories.
Statistical methods	12	(*a*) Statistical methods used in this study are described in the 7th paragraph of this session.
		(*b*) There are no subgroups in this study.
		(*c*) There was no missing data in this study
		(*d*) Not applicable
		(*e*) Not applicable
**Results**		
Participants	13*	(a) Number of participants is presented in the first paragraph of results session and detail socio-demographic characteristics in the table 1 too.
		(b) There was no non-participation during this study
		(c) This was cross-sectional study so; there is no flow as that of longitudinal study.
Descriptive data	14*	(a) Characteristics of study participants (eg demographic, clinical, social) and information on exposures and potential confounders is presented in tables 1 & 2
		(b) There was no missing data.
Outcome data	15*	Outcome variable is discussed and summarised under table 2 and figure 1.

Main results	16	(*a*) Unadjusted estimates and confounder-adjusted estimates and their precision (eg, 95% confidence interval) are presented in table 3.
		(*b*) Category boundaries for continuous variables were categorized and reported in all three tables.
		(*c*) We used odds ratio
Other analyses	17	Other analyses is not done
**Discussion**		
Key results	18	Key results to study objectives are discussed under discussion session with references.
Limitations	19	Limitations related to the current study are discussed in the final paragraph of discussion session on the way of viewing direction for researchers.
Interpretation	20	A cautious overall interpretation of results considering objectives, results from similar studies, and other relevant evidence is discussed under discussion session.
Generalisability	21	Generalisability (external validity) of the study results is pointed out under discussion and conclusion sessions.
**Other information**		
Funding	22	Information regarding the source of funding and the role of the funders for the present study is presented under acknowledgment session.
